# Insights into cancer risk from a tuberous sclerosis complex zebrafish model

**Published:** 2013-07

**Figure:**
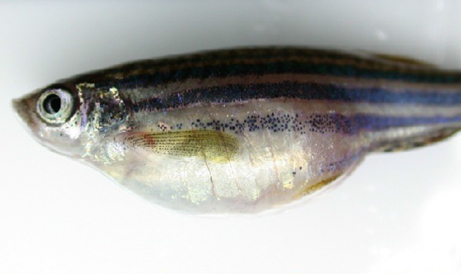


Tuberous sclerosis complex (TSC) is a rare genetic disorder associated with benign tumour formation. The disease is caused by loss-of-function mutations in either of the tumour suppressor genes *TSC1* or *TSC2*. In normal cells, the proteins encoded by these genes collectively inhibit mTORC1 (mechanistic target of rapamycin complex I) signalling; when absent, mTORC1 signalling is increased. Paradoxically, in other disorders characterised by augmented mTORC1 signalling, overt malignancies are observed. Ess and colleagues exploited this difference to gain mechanistic insights into tumorigenesis. They established a zebrafish model of TSC that develops cancer, by generating *tsc2;p53* compound mutants. Compared with *p53* single mutants, tumorigenesis and angiogenesis were enhanced in *tsc2;p53* zebrafish. Treatment with an mTORC1 inhibitor, rapamycin, inhibited tumour formation. This work provides *in vivo* evidence that cancer risk in p53-deficient individuals might be modulated by TSC1 or TSC2 mutations. **Page 925**

